# Forecasting imbalances of human resources for health in the Thailand health service system: application of a health demand method

**DOI:** 10.1186/s12960-018-0336-2

**Published:** 2019-01-08

**Authors:** Nonglak Pagaiya, Pudtan Phanthunane, Adun Bamrung, Thinakorn Noree, Karnwarin Kongweerakul

**Affiliations:** 1 Faculty of Public Health, Khonkaen University, 123 Mitraphap road, Muang, Khonkaen, 40000 Thailand; 2 Naresuan University, Muang, Phitsanulok, 65000 Thailand; 3 Khon Kaen Provincial Health Office, Muang, Khonkaen, 40000 Thailand; 4 International Health Policy Program, Ministry of Public Health, Tiwanon road, Muang, Nonthaburi, 11000 Thailand

**Keywords:** Human resources for health planning, Health demand method, Thailand

## Abstract

**Background:**

For an effective health system, human resources for health (HRH) planning should be aligned with health system needs. To provide evidence-based information to support HRH plan and policy, we should develop strategies to quantify health workforce requirements and supply. The aim of this study is to project HRH requirements for the Thai health service system in 2026. HRH included in this study were doctors, dentists, nurses, pharmacists, medical technicians (MTs), physiotherapists (PTs), and Thai traditional medicine (TTM) practitioners.

**Methods and results:**

The study mainly relied on the secondary data in relation to service utilization and population projection together with expert opinions. Health demand method was employed to forecast the HRH requirements based on the forecasted service utilizations. The results were then converted into HRH requirements using the staffing norm and productivity. The HRH supply projection was based on the stock and flow approach in which current stock and the flow in and out were taken into account in the projection. The results showed that in 2026, nurses are likely to be in critical shortages. The supply of doctors, pharmacists, and PTs is likely to be surplus. The HRH requirements are likely to match with the supply in cases of dentists, MTs, and TTM practitioners.

**Conclusion:**

In 2026, the supply of key professionals is likely to be sufficient except nurses who will be in critical shortages. The health demand method, although facing some limitations, is useful to project HRH requirements in such a situation that people are accessible to health services and future service utilizations are closely linked to current utilization rates.

## Introduction

Human resources for health (HRH) availability has improved in some countries. Still, shortages, skill-mix imbalances, maldistribution, barriers to inter-professional collaboration, rural turn over, and limited availability of health workforce data persist in many countries [1]. Recent efforts by the World Health Organization (WHO), the Global Health Workforce Alliance (GHWA), and partner organizations to facilitate the development of a global HRH strategy for the period 2016–2030 reflect the growing recognition of the importance of HRH planning. They also highlighted *that* “countries should build planning capacity to develop or improve HRH policy and strategies that quantify health workforce needs, demands and supply under different future scenarios” [1].

Human resources for health (HRH) planning is the process in which an organization attempts to estimate the HRH supplies and requirements; determine the most appropriate balance among the skills, distribution, and number of health workers; and make a plan to redress HRH challenges [2]. Effective HRH planning thus needs evidence-based information to support, so as HRH requirement and HRH supply projection can contribute quantitatively for the resilience HRH plan. Planning for HRH thus has a central role in order to continue progressing towards the realization of the sustainable development goals (SDGs).

Ranges of HRH projection approaches were made available; population ratio, health need, health demand, and service target methods and each of these approaches have their advantages and limitations [3]. The simple projection of *population ratio method*, although require less data, does not take into account the effects of changes in health service utilization and HRH performance [4]. *Health need method* explores changes in population needs for health services base on changes of patterns of health problems [3]. Compare to other methods, it is considered a comprehensive approach for HRH projection [4]; however, drawbacks include challenges in defining needs in terms of coverage and quality, and the projection may generate unrealistic number of HRH requirements without adjusting to mal-distribution [5]. *Health demand method* draws on observed service utilization rate, applies to future population profile, and converts to HRH requirements. The approach is appropriate for such health system that the population is accessible to a suitable mix of services. However, the approach requires considerable sets of data and the projection trends to base on status quo situation [5]. *The service target* approach sets targets for specific services using current service provision, other factors, and expert opinions [4]. This approach may be useful in planning critical health care services or service for specific population; however, the approach may depend on unreliable assumptions [4]. Taking all these aspects into consideration, this study employed health demand method to forecast HRH requirement for Thailand health service system because of the high health service coverage of Thailand health service [6] and the availability of administrative data system.

### Thailand health service system

Thailand is currently an upper middle-income country and is making progress towards meeting the SDGs. Although Thailand has made significant success in some health indicators (SDG 3), some other health challenges persist and need crucial attentions. The unfinished agenda of infectious diseases such as tuberculosis and HIV/AIDS coupling with non-communicable disease, such as cancer, cardiovascular diseases, diabetes mellitus, and hypertension, and traffic accident have threatened Thailand health system [7]. What is more, the increase of elderly health needs has prompted Thailand to improve health services to be in line with people needs.

With regard to the Thai health service system, virtually, everyone (99.84%) was covered by health insurance while other forms of social security have expanded [8]. Available health facilities delivering services to people can be classified according to three-tier service systems: primary, secondary, and tertiary care facilities [9]. *The primary health care facilities* are the first contacts which individuals, families, and communities have with the health care system. Services provided incorporate common illness treatment, health promotion, disease prevention, rehabilitations, and community health interventions. This type of health facilities includes health centers and private clinics without bed. *The secondary health care facilities* provide curative care with the initial referral being made by the primary care professionals. Care provision is more complex and need higher medical expertise. Such health facilities include the Ministry of Public Health (MoPH) community hospitals, other public hospitals, and private hospitals. *The tertiary health care facilities* provide specialized care, usually on referral from primary or secondary medical care personnel, by specialists working in a facility that has personnel and facilities for special investigation and treatment. These facilities include MoPH general and regional hospitals, university hospitals, other public hospitals, and private hospitals [9].

Approximately 54% of Thai population resides in rural areas where they are mainly served by health facilities responsible to MoPH [10]. These include 9777 health centers which cover 100% of all sub-districts and 780 community hospitals (range from 30 to 120 bed hospitals) covering 88.8% of all districts [8]. The primary health care facilities located at urban areas comprise MoPH community medical centers, municipality health centers, private medical clinics, and drug stores. In urban areas of 77 provinces, the secondary and tertiary medical services are provided by 177 MoPH hospitals, 11 medical school hospitals, 105 other public hospitals, and 249 private hospitals [8].

### HRH planning challenges

The density of doctors, nurses, and midwives of Thailand was 3.1/1000 population [11], well below the recommendation threshold of 4.45/1000 population [1], indicating the shortages of HRH. In addition, the maldistribution of HRH, particularly between rural and urban areas, hindered the progress of the Thai health system. It is well recognized that, to address the HRH challenges, the HRH requirements should be aligned with the health system needs in terms of the number, skills, and distribution. The evidence-based quantification of HRH requirements is thus required. Already several attempts have been made to estimate and forecast the HRH requirements for doctors [12], dentists [13], nurses [14], and pharmacists [15]; some challenges have been elucidated. The requirements were dated and each professional was projected individually without taking into account for team-based planning. Moreover, the requirements were mainly focused on public sectors which were likely to underestimate the country requirements. This study is therefore attempted to fill the gaps by forecasting HRH team in 2026 which comprise doctors, dentists, nurses, pharmacists, MTs, PTs, and TTM practitioners in primary health care, secondary health care, and tertiary health care services in Thailand.

## Methods

To forecast the HRH requirements, the approach mainly relied on the secondary data and stakeholder committee opinions. Since the available secondary data were mostly related to curative and rehabilitative service utilization, the health demand method or service utilization method was then used to project HRH requirements. It is suggested that this method is appropriate for the countries where service utilization and related data are available and people are accessible to health services [5]. The method was considered appropriate as this study focused mainly on HRH requirements relating to service utilization at 3 health facility settings: primary, secondary, and tertiary health facilities. The high health insurance coverage (99.84% of population) and low unmet health needs (1.5% of sick patients) [6] have justified the utilization of the health demand method. HRH professionals included in the forecast are HRH whose main functions are related to curative and rehabilitative services including doctors, dentists, nurses, pharmacists, MTs, PTs, and TTM practitioners.

Health demand method forecasts the HRH requirements based on the forecasted service utilizations and then converts to HRH requirements by the staffing norm and productivity as the following steps.

1. Set up a technical working group (TWG) comprised of 13 members who have particular knowledge and expertise in service delivery. To ensure all concerned professionals involved, 7 representatives from 7 professional councils (medical, nursing, dentist, pharmacist, MT, PT, TTM practitioner councils), 2 representatives from primary care facilities, 2 representatives from secondary care facilities, and 2 representatives from tertiary care facilities were included into the TWG members. The TWG met regularly to provide technical input and expert opinions at each step of the projection process.
2. Identify main services provided by each professional at each particular level of care. Based on services provided by health facilities under MoPH, researchers reviewed main services and presented to the TWG to discuss and approve main service to be included. At the primary care level, main services included were outpatient, dental, drug dispensing, and rehabilitation services. At the secondary and tertiary care level, main services consisted of services in relation to outpatient (OP), inpatient (IP), surgery, birth delivery, accident and emergency (A&E), intensive care unit (ICU), ultra sound, drug dispensing, dental care, laboratory, and TTM services (Table 1). Outpatient services at the primary care, for instance, were provided by doctors, dentists, nurses, and TTM practitioners, while outpatient services at the secondary and tertiary care facilities were provided by doctors, dentists, nurses, pharmacists, MTs, PTs, and TTM practitioners.
  Different service activities were provided differently by each professional, according to their professional roles and functions. The proportion of work allocations among health workforce team have been decided among TWG based on the expert experience. Due to unavailability of doctors at the rural primary care, doctors provided treatment services for about 20% of outpatients at public facilities, and 100% of outpatients services were provided by nurses. However, doctors provided services to all outpatient services at private clinics. At the secondary and tertiary health facilities, 100% of outpatient services are provided by both doctors and nurses. Pharmacists dispensed at about 20% of outpatients at primary care, and 70% of outpatient visits and inpatient days at secondary and tertiary care levels required drug dispensing service. All dental services at all 3 levels of care were provided by dentists. Due to unavailability of PTs and MTs at primary care, physiotherapy and laboratory services were only provided at secondary and tertiary care facilities. According to the Department of Thai Traditional Medicine [16], TTM service clients were estimated to 17.5% of general outpatients and 70% of this figure needed Thai traditional interventions (see details in Table 1).
3. Collect the service utilizations and related data to project the service utilizations in the next 10 years (2026), and these steps were followed:
  1. Collect and collate the MoPH administration data relating to service utilizations at 3 levels of care. Since the 2013 data provided details of annual volume of main services provided at each level of care and was verified by MoPH committee [17], the TWG decided to use the service utilization and related data produced in 2013 for the purpose of HRH projection.
  2. MoPH service utilization proportion by age and sex was used to compare with the country population of the same age and sex in 2013 [18]. The derived proportion was then used to multiply the projected population in 2026 of the same age and sex in order to obtain the projected service utilization at each level of care in 2026. By this approach, it was under the assumption that the pattern of service utilizations would remain constant in the future.
  3. Use the percentage of service utilization by health facility types (MoPH, other public and private facilities) obtained from the survey report of the National Statistical Office [6] to add on the volume of the MoPH service utilization. Of all outpatient visits, 70, 24, and 6% of them used the services at MoPH facilities, private facilities, and other public facilities, respectively. For in-patient utilization, 76, 16, and 8% of all inpatients used the services at MoPH facilities, private facilities, and other public facilities, respectively. Regarding dental services, the unmet need was 1.05%, and service utilization at private and other public sectors were 41.44% and 4.41%, respectively. To obtain overall service utilization of the country, the service utilization of other public facilities and private facilities is added on to those of the MoPH’s. In addition, to ensure the unmet needs have been covered, the unmet needs of 1.5% of outpatients and 0.14% of inpatients [6] were topped up.
4. Identify work productivity and workload of each professional at each level of care. Time requirement per service of each professional was obtained from the MoPH information system [17] and presented to TWG for the consensus of expert opinions. Service hour per case of doctors were 0.12, 0.25, 0.25, and 2.5 h for outpatient, A&E, ultrasound, and surgery services, respectively. Nursing time services for outpatient, A&E, ultrasound, and surgery services were 0.2, 0.6, 1, and 4 h/ case, respectively. As IP and ICU patients needed 24-h services, doctors spent 0.33 h and 1 h per patient day and nurses spent 3.5 h and 12 h per patient day, respectively. Surgery service carried out at secondary and tertiary facilities required a team of 1 and a half doctors and 2 and a half nurses per case. Normal delivery service required 2 nurses and abnormal delivery service required 2 and a half nurses for each case. Pharmacy service included was limited only to drug dispensing service and took 0.08 h per prescription. Dental service time requirement ranged from 0.47to 3 h per service. PT spent 0.8 h for a case. TTM practitioner took 0.12 and 1 h per case for diagnosis and Thai traditional intervention, respectively. Ranges of laboratory services carried out by MTs took about 0.02–0.33 h per test (see more details in Table 1).
5. Calculate workload by multiplying the number of annual services of the year 2013 by the time spent per service by each professional at each level of care. Doctor, for instance, carried out 110 290 828 outpatient services at secondary care and doctor workloads were calculated equal to 13 234 899 man hours (110 290 828 × 0.12 h). The calculation at each level of care in the year 2013 and 2026 were carried out using the same approach.
6. Convert workload into HRH requirements. The TWG used the staff norm set by the MoPH [17] in which 1 full-time equivalent (FTE) equaled 1680 working hours per year (working 7 h a day and 240 days a year) to convert workload into HRH requirements. Doctor requirements for outpatient service, for instance, were obtained from dividing doctor outpatient workload, 13 234 899 man hours by 1 FTE (1 680 working hours per year); the number of doctor requirements for outpatient service was thus 7 878 FTE doctors.
7. Determine the time spent for other services/activities. Although workload included in this study was mainly curative and rehabilitation services, HRH daily functions should include other important aspects, such as home health care services, health promotion services, disease prevention and public health interventions, administrative work, meeting, leave, and capacity building. Therefore, based on the time motion study of Pagaiya et al. [19], the TWG reviewed and decided the allowance level to accommodate other necessity activities of HRH. At secondary and tertiary care, the allowance across all professionals, except pharmacists, were 15% of their FTE. Since pharmacist’s main activities consist of drug dispensing, drug/medical supply production, and drug and medical supply management, the proportion of their time spent for drug dispensing was approximated at about 56%. The pharmacist allowance was therefore agreed at 44%. At primary care, nurses, dentists, pharmacists, and TTM practitioners have to carry out outreach activities at the community and some other administrative works; the allowance proportion allocated for other work was agreed at 30%.
  After converting workloads into the HRH requirements, the allowance proportion was toped up at each professional. Doctor requirements at primary care was 7275 FTE; for instance, the requirements after addition of 15% allowance was 8366 FTE.
  In the projected scenario in 2026, it was presumed that all planning variables except population are held constant at current levels.
8. Match the projected HRH requirements with the projected HRH supply. The supply projections were reviewed from the 7 professional requirement and supply studies [20–26]. The stock and flow model was used for each study in order to project the HRH supply in 2026 [11]. The current stock of each professional was obtained from the licensed HRH who were actively practicing. The potential inflow into the HRH stock was based on the production plan of each professional, while the outflow against the stock was defined according to the potential number of retirements, death, resignation from the health system, and drop out from colleges/universities. As the emigration and immigration of HRH was minimal, so this information was not taken into account for the forecast. The projected HRH supply was then calculated and matched with the projected requirements to identify the HRH imbalance.

**Table 1:** Main services, work productivity, and proportion of work allocation by professionals and level of care

Professionals	Main services	Primary care		Secondary and tertiary care	
		Proportion of workload (%)	time/service (hours)	Proportion of workload (%)/number	time/service (hours)
Doctors	OP Treatment	20%	0.12	100%	0.12
	IP treatment	–	–	100%	0.33
	Surgery	–	–	100%	2.5^a^
	Normal delivery	–	–	100%	0.25
	Abnormal delivery	–	–	100%	2.5
	A&E services	–	–	100%	0.25
	ICU services	–	–	100%	1
	Ultrasound services	–	–	100%	0.25
Nurses	OP Treatment	100%	0.2	100%	0.2
	IP treatment	–	–	100%	3.5
	Surgery	–	–	100%	4^a^
	Normal delivery	–	–	100%	7^a^
	Abnormal delivery	–	–	100%	4^a^
	A&E services	–	–	100%	0.6
	ICU services	–	–	100%	12
	Ultrasound services	–	–	100%	1
Pharmacists	OP Dispensing	20%	0.08	70%	0.08
	IP Dispensing	–	–	70% of patient days	0.08
Dentists	General services	100%	0.47	100%	0.47
	Special services	–	–	100%	2
	IP services	–	–	100%	3
Physiotherapists	PT services	100%	0.8	100%	0.8
Thai traditional practitioners	Diagnosis	17.5% of OP	0.12	17.5% of OP	0.12
	Thai traditional treatment	70% of above	1	70% of above	1
Medical Technicians	Specimen collection	–	–	100%	0.02
	Hematology services	–	–	100%	0.15
	Microscope services	–	–	100%	0.17
	Clinical chemistry services	–	–	100%	0.03
	Immunology services	–	–	100%	0.1
	Microbiology services	–	–	100%	0.33
	Molecular biology and blood services	–	–	100%	0.18

^a^Operation carried out at secondary and tertiary services require 1.5 doctors and 2.5 nurses per case. Normal delivery requires 2 nurses and abnormal delivery require 2.5 nurses for each case

## Results

### HRH requirements in 2013

At primary care, only outpatient services were included in the HRH workload. The total outpatient services were 210 876 061 visits. Of this figure, 74 587 669 visits took place at private facilities in 2013. Doctor outpatient services were 27 257 679 visits at public facilities and 74 587 669 visits at private facilities. The workload calculation was equal to 12 221 441 working hour and that made 7275 doctor requirements. Nurses provided services to all 210 587 669 visits, and the conversion of nursing workload resulted in 25 104 nurse requirements. Drug dispensing provided by pharmacists for 42 175 212 visits was converted to 2092 pharmacist requirements. Dentists delivered 10 170 917 general dental services, and 2825 dentists were required for providing such dental care services at primary care facilities. The TTM services provided at primary health care facilities was 29 438 293 services, and that required 4673 TTM practitioners. When 30% allowance for outreach and other activities at primary health care facilities were added up for all professionals, excepted doctor which only 15% of allowance was topped up, the requirements for doctors, nurses, pharmacists, dentists, and TTM practitioners are shown in Table 2.

**Table 2:** Workload and HRH requirements at primary health care services in 2013

Professionals	No. of services	Man hours	HRH requirements (FTE)	
			FTE	Include allowance
Doctors				
Public facilities	27 257 679	3 270 921	1947	8366
Private facilities	74 587 669	8 950 520	5328	
Nurses	210 876 061	42 175 212	25 104	32 635
Pharmacists	42 175 212	3 514 601	2092	2720
Dentists	10 170 917	4 746 428	2825	3249
TTM Practitioners	29 438 293	7 850 211	4673	6075

The services provided and HRH requirements at secondary and tertiary care facilities are shown in Tables 3 and 4. The quantity of services provided by doctors and nurses at secondary care were dominated by OP services, IP services, A&E services, ultrasound services, and normal and abnormal delivery services at approximately 110 290 828 visits, 12 949 845 patient days, 5 974 225 visits, 902 553 visits, and 281 048 cases and 15 664 cases, respectively. Comparing to those of the secondary care, although OP services at tertiary care facilities were about a half, the IP services was double, particularly those of the ICU service which were about 19 times more. The number of surgical cases was almost 13 times higher, and the abnormal delivery cases were about 3 times higher. All other services were comparable. Drug dispensing at tertiary care was slightly higher than those at secondary care. Laboratory services provided were about 3 times as high as those at secondary care. Clinical chemistry, microbiology, molecular biology, and blood banking tests/services were greatly higher at tertiary care. Details are shown in Table 3.

**Table 3:** Workload and requirements of doctors, nurses, pharmacist, and MT by level of care in 2013

Professionals/services	Secondary care			Tertiary care		
	No. of services	Man hours	HRH requirements (FTE)	No. of services	Man hours	HRH requirements (FTE)
Doctors						
OP treatment	110 290 828	13 234 899	7878	62 206 113	7 464 734	4443
IP treatment	12 949 845	4 273 449	2544	21 046 774	6 945 435	4134
Surgery	106 549	399 557	238	1 352 510	4 463 283	2657
Normal delivery	281 048	70 262	42	243 857	60 964	36
Abnormal delivery	15 664	39 160	23	43 368	95 409	57
A&E services	5 974 225	1 493 556	889	4 851 152	1 212 788	722
ICU	125 683	125 683	75	2 386 955	2 386 955	1421
Ultrasound services	902 553	225 638	134	970 912	242 728	144
Total (include 15% allowance)			13 596			15 657
Nurses						
OP Treatment	110 290 828	22 058 166	13 061	62 206 113	12,441,2239	7405
IP treatment	12 949 845	45 324 456	37 187	21 046 774	94 710 483	56 375
Surgery	106 549	1 065 487	874	1 352 510	12 781 220	7608
Normal delivery	281 048	3 934 676	3228	243 857	3 414 004	2032
Abnormal delivery	15 664	156 639	129	43 368	409 824	244
A&E services	5 974 225	3 584 535	2122	4 851 152	2 522 599	1502
ICU	125 683	1 508 195	1237	2 386 955	28,643,4579	17 050
Ultrasound services	902 553	902 553	741	922 366	922 366	549
Total (include 15% allowance)			53 759			106 680
Pharmacists						
Drug dispensing	68 346 611	5 695 551	4857	82 958 564	6 913 214	4115
Total (include 44% allowance)			6995			5926
MT						
Specimen collection	16 076 429	267 940	159	10 058 590	167 643	100
Hematology	15 897 187	2 384 578	1419	18,391,769	2,758,765	1642
Microscope	9 857 133	1 642 855	978	7,898 760	1 316,460	784
Clinical chemistry	76 216 024	1 905 401	1134	101 608 462	2 540 212	1,512
Immunology	6 526 278	652 628	388	8 350 144	835 014	497
Microbiology	2 377 038	792 346	472	6 433 612	2 144 537	1277
Molecular biology and blood banking	842 625	154 037	92	6 893 524	1 259 161	750
Total (include 15% allowance)			5339			7546

**Table 4:** Workload and requirements of dentists, PT and TTM practitioners by level of care in 2013

Professionals/services	Secondary care			Tertiary care		
	No. of services	Man hours	HRH requirements (FTE)	No. of services	Man hours	HRH requirements (FTE)
Dentists						
General	16 695 081	7 791 038	4638	5 442 507	2 539 836	1512
Special	1 679 730	3 359 460	2000	1 244 250	2 488 500	1481
IP services	8981	26 944	16	14 279	42 837	25
Total (include 15% allowance)			7651			3471
Physiotherapists						
PT services	4 244 360	2 979 466	1773	3 801 182	2 805 212	1670
Total (include 15% allowance)			2040			1920
TTM practitioners						
Diagnose	19 311 924	4 827 981	2874	10 892 290	2 723 073	1621
TTM interventions	9 655 962	7 241 971	4311	5 446 145	4 084 609	2431
Total (include 15% allowance)			8262			4660

Dental services, PT services, and TTM services were higher at secondary care facilities, compared to those at tertiary care facilities. Dental services at secondary care services were 3 times as high as those of tertiary care services and the PT services at secondary care were slightly higher than those at tertiary care facilities. TTM services provided at secondary care facilities were about 1.8 times higher than those at the tertiary care. Details are shown in Table 4.

When working hour was conversed into HRH requirements and topped up with allowance percentage (44% for pharmacists and 15% for all other professionals), the secondary care health system required 13 596 doctors, 53 753 nurses, 6995 pharmacists, 5339 MTs, 7651 dentists, 2040 PTs, and 8262 TTM practitioners. The HRH requirements for tertiary care were 15, 576 doctors, 106 680 nurses, 5926 pharmacists, 7546 MTs, 3471 dentists, 1920 PTs, and 4660 TTM practitioners.

### HRH requirements in 2026

The projection of service utilization and HRH requirements in 2026 are shown in Tables 5 and 6. The amount of OP services in 2016 will be the highest at the primary care compared to the secondary and the tertiary care. IP services are highest at the tertiary care; particularly, ICU services will be approximately 19 times of those of the secondary care. Surgery, abnormal delivery and ultrasound services will be also performed highest at the tertiary care. In contrast, A&E services will be higher at the secondary care facilities. Accordingly, laboratory services will be higher at the tertiary care. The volume of drug dispensing at the secondary care will be higher compared to that at the tertiary or the primary care. Details are given in Table 5.

**Table 5:** Workload and requirements of doctors, nurses, pharmacist, and MT by level of care in 2026

Professionals/services	Primary care		Secondary care		Tertiary care	
	No. of services	HRH requirements (FTE)	No. of services	HRH requirements (FTE)	No. of services	HRH requirements (FTE)
Doctors						
OP Treatment	103 902 454	7422	121 424 710	8673	68 490 610	4892
IP treatment			14 366 573	2822	23 399 074	4596
Surgery			118 205	264	1 503 674	2954
Normal delivery			311 795	46	271 112	40
Abnormal delivery			17 378	26	48 215	63
A&E services			6 577 325	979	5 341 249	795
ICU care			139 433	83	2 653 734	1580
Ultrasound services			997 480	148	1 074 213	160
Total (include allowance)		8535		14 998		17 342
Nurses						
OP Treatment	221 161 598	22 839	121,424 710	14 455	68 490 610	8154
IP treatment			14 366 573	29 930	23 399 074	62 676
Surgery			118 205	704	1 503 674	8458
Normal delivery			311 795	2598	271 112	2259
Abnormal delivery			17 378	103	48 215	271
A&E services			6 577 325	2349	5 341 249	1653
ICU care			139 433	996	2 653 734	18 955
Ultrasound services			997 480	594	1 074 213	607
Total (include allowance)		29 691		59 489		118 488
Pharmacists						
Drug dispensing	44 232 320	2165	107 983 815	5356	91 762 863	4552
Total (include allowance)		3118		7713		6555
MT	–	–				
Med Lab. services			141 233 405	5131	176 619 399	7259
Total (include allowance)				5901		8348

**Table 6:** Workload and requirements of dentists, PT, TTM practitioners by level of care in 2026

Professionals/services	Primary care		Secondary care		Tertiary care	
	No. of services	HRH requirements (FTE)	No. of services	HRH requirements (FTE)	No. of services	HRH requirements (FTE)
Dentists						
Dental services	11 188 009	3108	20 222 171	7319	8 588 238	3973
Total (include allowance)		3574		8416		4569
Physiotherapists	–	–				
PT services			4 690 764	1960	4 205 613	1847
Total (include allowance)				2254		2124
TTM practitioners						
TTM services	31 659 908	5026	31 892 200	7910	17 989 059	4462
Total (include allowance)		6534		9096		5131

Dental services will be highest at the secondary care compared to the primary or tertiary care. PT services at the secondary care will be close to those at the tertiary care. TTM services will be high at the primary and the secondary care compared to the tertiary care. Details are given in Table 6.

The projection of HRH requirement in 2026, after inclusion of allowance percentage, revealed that the health service system at the primary care facilities will require 8535 doctors, 29 691 nurses, 3118 pharmacists, 3575 dentists, and 6534 TTM practitioners. The secondary health facilities will require 14 998 doctors, 59 489 nurses, 7713 pharmacists, 5901 MT, 8416 dentists, 2254 PTs, and 9096 TTM practitioners. The projected tertiary care services will require 17 342 doctors, 118 488 nurses, 6555 pharmacists, 8348 MT, 4569 dentists, 2124 PTs, and 5131 TTM practitioners.

### Sensitivity analysis

The effective HRH requirements is dependent not only on the number of service utilization but also on the average time spent on each service, working hour, proportion of time spent on other activities, etc. Time spent on each service or productivity of each professional varies across health facilities including public and private sectors or even within health facilities. The range of time spent on each service, i.e., the baseline time and the baseline time ± 10%, were chosen to perform sensitivity of various scenarios, while other factors held constant. Table 8 demonstrates the sensitivity of HRH requirements to different level of time spent per service. The higher the time spent per service, the higher the number of HRH requirements.

### HRH supply projection in 2026

The HRH supply projection was derived from the professional studies of doctors [20], nurses [21], dentists [22], pharmacists [23], MTs [24], PTs [25], and TTM practitioners [26], for which the stock and flow method was used. The number of doctors existing in 2013 was 41 746, and the ratio per 1000 population was 0.65. The flow in data derived from the newly graduates of all medical schools were 3121 annually. The doctor flow out was approximately 1% loss rate from death and retirement and approximately 25% of registered doctors do not engage in clinical practice. The doctor supply in 2026 is projected to 62 779 doctors. The existing nurses in 2013 were 158 317, and the newly entrants of nursing students are about 10 000 nurses per year. Flow out was calculated from 4.44% loss rate due to change of jobs, retirements and deaths, and 5% drop out rate of nursing students. The projection of nurses in 2026 is estimated to 180 992 nurses.

Pharmacist stock was 24 940 in 2013, and the production plan is approximately 1700 pharmacists per year. Taking into account of 2% loss rate per year, the pharmacist projection is amounted to 38 905 in 2026. Current dentist stock was 10 506 in 2013. With 825 dentist production per year and 1.5% loss rate, the projection of dentists is estimated to be 18 675 in 2026.

The number of PTs existing in 2013 was 4301, and the annual student entrants are 1155 per year. Loss rate of PTs comprised of 2% from death and retirement, 20% of dropout rate of PT students, and 5% of those not engage in PT services. Taking into account flow in and flow out rates, the PT projection in 2026 is estimated to be 9736. The number of TTM practitioners existing in 2013 was 9224. With 1080 TTM practitioner graduates per year and 2% loss rate from death and retirement, the projection of TTM practitioners in 2026 is 19 080. In 2013, the existing MTs were 14 047. The newly graduates of MTs per year are 911 and the loss rate is 5% per year. The MT projection in 2026 is estimated to be 16 078. See details in Table 7.

**Table 7:** Current and projected HRH supply by professional

Professionals	Current HRH (2013)		Production/year	Loss rate/year	Projected supply (2026)
	number	Population ratio(/1 000)			
Doctors	41 746	0.65	3121	− 3% drop out from medical students − 1% loss from death and retirement − 25% of registered doctors not involve in clinical practice	62 779
Nurses	158 317	2.45	10 000	− 5% drop-out rate of nursing students − 4.44% loss from change job, death and retirement	180 992
Pharmacists	24 940	0.38	1700	2% loss from death and retirement	38 905
Dentists	10 506	0.16	826	1.5% loss from death and retirement	18 675
PT	4301	0.07	1155	− 20% drop out rate of PT students − 2% loss from death and retirement − 5% not involve in PT services	9736
TTM practitioners	9224	0.14	1080	2% loss from death and retirement	19 080
MT	14 047	0.22	911	5% loss from death and retirement	16 078

### Matching HRH requirements and supplies in 2026

Table 8 illustrates the comparison between HRH requirements and supplies, and the gap identification in 2026. Nurses are likely to be in critical shortages, since there is a requirement of about 186 901–228 435 nurses, but the number of nurses supplied is projected to be 180 992. Some professionals’, for instance, doctors, pharmacists, and PTs, supply are likely to be surplus at about 17 818–25 993 doctors, 19 780–23 258 pharmacists, and 4920–5796 PTs. In cases of dentists, MTs, and TTM practitioners, the HRH requirements are likely to match with the HRH supply.

**Table 8:** HRH requirements VS supply in 2016

	HRH requirements in 2026		HRH supply in 2026	Shortages/surplus
	Baseline time	Baseline time ± 10%		
Doctors	40 875	36 786–44 961	62 779	17 818 to 25 993
Nurses	207 668	186 901–228 435	180 992	− 5909 to − 47 443
Pharmacists	17 386	15 647–19 125	38 905	19 780 to 23 258
Dentists	16 559	14 904–18 215	18 675	461 to 3772
Physiotherapists	4 378	3940–4816	9 736	4 920 to 5796
TTM practitioners	20 761	18 685–22 837	19 080	− 3,757 to 395
MT	14 249	12 824–15 674	16 078	404 to 3254

## Discussion

Although various approaches have been carried out to increase the accuracy in projecting the future HRH requirements and supplies, this kind of study often faces some limitations. First, several factors critically affecting demand of health care are not taken into account in the projection. Challenges facing Thai health system including rapid increase of aging population, increase of people living with chronic conditions, and growing demand of services provided in home and community settings should be included in the projection to show the direction towards which the health service system and HRH situation may be heading. Second, service utilization data used were focused mainly on curative and rehabilitation services. Health services in relation to health promotion, disease prevention, and home health care were not taken into account. Third, the HRH requirements did not cover HRH working as administrators, lecturers, researchers, and business related to health. The projected HRH requirements in the study therefore were likely to be underestimated. Fourth, the study mainly used the secondary data from various sources in projecting HRH requirements and supplies. Since the quality of data was varied among the sources, the results have some risks in the data accuracy and integrity. Fifth, the HRH requirements varied upon the volume of service utilization, time spent on each service, working hour, and proportion of time spent on other activities. The fact that the changes of these figures critically affect the number of HRH requirement, the results should be viewed with considerations. Sixth, the TWG play important role on crucial figures affecting HRH requirements. Despite the fact that several key stakeholders involved in decision making, the results risk towards professional bias and likely to require more HRH than the health system can afford.

Despite some limitations, the present results demonstrated the potentials to project HRH requirements in a multi-professional manner rather than the single professional projection to consider the possible interaction and team work, particularly task sharing of doctors and nurses at the primary care level. The results support the recommendation made by Ono et al. [27] in that the HRH projection should shift from uni-professional to multi-professional approach. Moreover, in this study, multi-professional committees were involved at all important process of the HRH projection. This multi-professional projection approach has been carried out also in other studies, i.e., the calculation of family doctors, nurses, public health workers, and others at community health centers in China [28]; the projections of doctor, nurse, and midwife requirements in OEDC [29]; and the projection of skill-mix among doctors, advanced practice nurses, and physician assistants in Finland, the Netherland, Switzerland, and the United States of America [27].

The present results illustrated that, in 2026, doctors, pharmacists, and PTs were likely to be surplus, if the service system and HRH system be carried out as usual. On the other hand, the number of nurses will be in extremely shortage. The supply and requirements of dentists, MT, and TTM practitioners will be in balance. Comparing with the threshold of 4.45 (doctors + nurses + midwives)/1000 population to achieve the median score (25%) of attainment of 80% coverage for the 12 selected SDG tracer conditions [1], the requirement of doctors and nurses in this study was about 3.64/1000 population, slightly below the recommendation.

However, the HRH requirements in this study were likely to be underestimated. First, doctors and pharmacists provided care at about 20% of outpatient visits at public primary care facilities, and thus, the requirements of doctors and pharmacists were lower than what should be. Second, home health care for aged and chronically ill patients were not included in the scenario, so that the requirement of home health care providers, i.e., PTs, doctors, and nurses, were thus underestimated. Third, if drug industries and other drug-related functions were included in the projection, the requirements of pharmacists would be much higher. HRH requirements applying the service utilization for multi-professional approach in this study were lower than those used in different methods for single professional projections in Thailand. Dentist requirements in 2026 by the service target method by Jaichuen [18] showed the needs with the range of 19 677–20 955 which were higher than the supply. Pharmacist projection by the system dynamic approach required 47 787 pharmacists in 2026 [19]. Sawaengdee [17] combined service utilization and health need methods to project the nurse requirement which showed similar results to this study. At the same time, PT requirement by the combination of service utilization and service target approaches showed the requirements of 7855–8378 [21] which was higher than that of the results of this study.

Although the results of this study indicated that the production of key professional is likely to be sufficient, the concerned issues are the absorption, distribution, and retention of HRH which were not under the scope of this study.

There are some advantages of the use of health demand methods in projecting HRH requirements. This approach is appropriate for the countries where the population is accessible to a suitable mix of health services and future service utilization rates are closely linked to current utilization rates. The approach used in this study has already been used in several studies to project HRH future requirements, particularly for curative services. The health demand method was used to project for future doctor requirements in Mexico [30], Australia, Belgium, and Canada [27] and dentist requirements in Australia [31]. Moreover, the approach has been used at sub-national level, for example, there was an attempt to calculate the requirements for doctors, nurses, public health workers, and allied health workers working at community health center in a district in Beijing, China [28].

## Conclusion

The results of this study implied that the production of key professional is likely to be sufficient in the future, except nurses which are likely to be in critical shortages. Health demand method used to project HRH requirements in this study, though there are some limitations, was likely to be applicable for countries where health service utilization constantly changed and was useful for micro-planning at the facility level. However, HRH planning should be reviewed and updated often as the context affecting health service is dynamic.

## Abbreviations

A&E: Accident and emergency
FTE: Full-time equivalent
HRH: Human resources for health
ICU: Intensive care unit
IP: Inpatient
MoPH: Ministry of Public Health
MT: Medical technician
OP: Outpatient
PT: Physiotherapist
SDGs: Sustainable development goals
TTM: Thai traditional medicine
